# Household, maternal, and child related determinants of hemoglobin levels of Ethiopian children: hierarchical regression analysis

**DOI:** 10.1186/s12887-019-1476-9

**Published:** 2019-04-15

**Authors:** Shimels Hussien Mohammed, Tesfa Dejenie Habtewold, Ahmad Esmaillzadeh

**Affiliations:** 1 Department of Community Nutrition, School of Nutritional Sciences and Dietetics, Tehran University of Medical Sciences-International Campus, Tehran, Iran; 2 Department of Epidemiology, University Medical Center Groningen, University of Groningen, Groningen, The Netherlands; 3 Obesity and Eating Habits Research Center, Endocrinology and Metabolism Molecular Cellular Sciences Institute, Tehran University of Medical Sciences, Tehran, Iran; 4 Department of Community Nutrition, School of Nutritional Sciences and Dietetics, Tehran University of Medical Sciences, Tehran, Iran; 5 Food Security Research Center, Department of Community Nutrition, Isfahan University of Medical Sciences, Isfahan, Iran

**Keywords:** Hemoglobin status, Anemia, Risk factors, Children

## Abstract

**Background:**

Anemia remains a major public health problem among children under five years old in Ethiopia, rising unexpectedly from 44% national prevalence in 2011 to 57% in 2016. In this study, we investigated the household, maternal and child-related dietary and non-dietary factors associated with hemoglobin (Hb) level of infants and young children.

**Method:**

We analyzed data from a nationally representative sample of 2902 children aged 6–23 months, included in the 2016 Ethiopian demographic and health survey (EDHS). Hierarchical linear regression analysis was done to identify the factors associated with Hb level. We reported adjusted β (aβ) with 95% confidence interval (CI).

**Result:**

Overall, 72% of children under 2 years of age were anemic in Ethiopia in 2016. Household factors: rich household wealth category (aβ = 0.48, 95%CI = 0.33–0.63, *P* < 0.001), and agrarian regions (aβ = 0.64, 95%CI = 0.40–0.88, *P* < 0.001) were significantly associated with a higher mean Hb level. Maternal factors: secondary and above education level (aβ = 0.69, 95%CI = 0.23–1.16, *P* = 0.004), and being not anemic (aβ = 0.40, 95%CI = 0.26–0.53, *P* < 0.001) were significantly associated with a higher mean Hb level. Child factors: age below 12 months (aβ = 0.72, 95%CI = 0.57–0.88, *P* < 0.001), female sex (aβ = 0.16, 95%CI = 0.03–0.30, *P* = 0.019), being not underweight (aβ = 0.22, 95%CI = 0.02–0.42, *P* = 0.031), average birth size (aβ = 0.25, 95%CI = 0.08–0.42, *P* = 0.003), no history of recent infection (aβ = 0.18, 95%CI = 0.02–0.33, *P* = 0.025), currently breastfeeding (aβ = 0.28, 95%CI = 0.12–0.44, *P* = 0.002), vitamin A supplementation (aβ = 0.17, 95%CI = 0.06–0.28, *P* = 0.021), and frequent meal feeding (aβ = 0.11, 95%CI = 0.05–0.16, *P* = 0.034) were significantly associated with a higher mean Hb level.

**Conclusion:**

Hb level was associated with various dietary and non-dietary influences originating from household, maternal, and child levels. A comprehensive approach, addressing the multi-factorial nature of Hb status, might stand an important consideration to reverse the recent rise in anemia prevalence in Ethiopia.

## Background

Anemia, marked by a low hemoglobin (Hb) level, continues to be a significant public health concern affecting almost a third of the world’s population. Infants and young children are of particular concern, developing anemia at a higher rate and bearing the highest burden [1]. In 2016, anemia prevalence among children under five years old in Ethiopia was 57%, rising unexpectedly from 44% in 2011 [2]. Infants and young children bear the highest burden of anemia in Ethiopia, with a 72% prevalence of anemia among those under two years of age [2]. The World Health Organization (WHO) classifies anemia prevalence above 40% as a severe public health problem [3].

Anemia is a multi-causal problem with a number of dietary and non-dietary risk factors [1, 4]. Food items with high phytate and polyphenol contents are associated with a high risk of anemia. Inadequate dietary or supplemental intake of iron, folate, and vitamin A often leads to anemia [4, 5]. While iron deficiency has long been considered the single greatest factor contributing to anemia, accounting for almost 50% of anemia globally [4, 5], recent reports suggest that iron deficiency is not as significant a culprit as was once thought [6]. Its contribution is particularly low in countries with high anemia and inflammation burdens, where it is estimated to account for 14 and 20% of the burden of anemia among preschool children, respectively [6]. Intestinal parasites, malaria, and infection are also among the main immediate causes of anemia, particularly in developing countries [4, 6]. Chronic illness or inflammatory conditions increase expression of hepcidin hormone, which reduces the absorption of iron by enterocytes and its exportation by ferroportin, thereby increasing the risk of anemia [7]. The main underlying conditions leading to anemia in developing countries are suboptimal feeding, caring and hygiene practices, coupled with poor health care. Poor socioeconomic status is one of the basic determinants of anemia [5, 8, 9].

Reducing the burden of anemia is one of the six global nutrition targets outlined by the WHO for the period 2012–2025 [10]. In Ethiopia, some interventions have been put in place to address the burden of anemia. These include distribution and promotion of the use of insecticide-treated mosquito nets, deworming and iron supplementation, and school- and community-based nutrition interventions [11]. While some studies are available on the determinants of anemia or Hb level in Ethiopia, most studies did not account for the hierarchical nature and interrelationships among the multilevel determinants [12, 13]. Their estimates were mainly based on single model regression analyses, which could be problematic. For example, the distal determinants of Hb level, like community and household factors, influence not only Hb level directly but also its underlying and proximal determinants like breastfeeding and dietary practices. Thus, including all variables in one model, a practice in most of the existing studies, may nullify or weaken the relation of the distal factors with Hb level [14]. Besides, given the recent increase in the prevalence of anemia in Ethiopia [2] and the time-varying nature of the contextual determinants, it stands timely and necessary to further investigate the determinants of Hb level. We used Hb level on a continuous scale to avoid the problem of potential statistical power loss due to dichotomization into anemic and non-anemic groups [15]. The use of Hb level on a continuous scale also enables to evaluate the relation of the determinant factors with the full spectrum of Hb level, not just with the state of anemia. Thus, in this study, we aimed to investigate the various household, maternal, and child-related dietary and non-dietary factors influencing Hb level of Ethiopian children aged 6–23 months using the latest nationally representative demographic and health survey, EDHS 2016.

## Methods

### Data source, study setting, and population

We used the dataset of children included in the EDHS 2016. EDHS is part of the international demographic and health survey (DHS) program, led by the United States Agency for International Development (USAID), in collaboration with other organizations and host countries [16]. In Ethiopia, the DHS has been conducted every five years since 2000. The latest survey was conducted in 2016 [2]. The full data set of EDHS 2016 is available and accessible on the DHS program website: http://dhsprogram.com/data/dataset/Ethiopia_Standard-DHS_2016.cfm. The survey was designed to be representative at both national and regional levels [2]. Children 6–23 months of age, with Hb level record, were included in this work.

### Sample size and sampling methodology

**Fig. 1:**
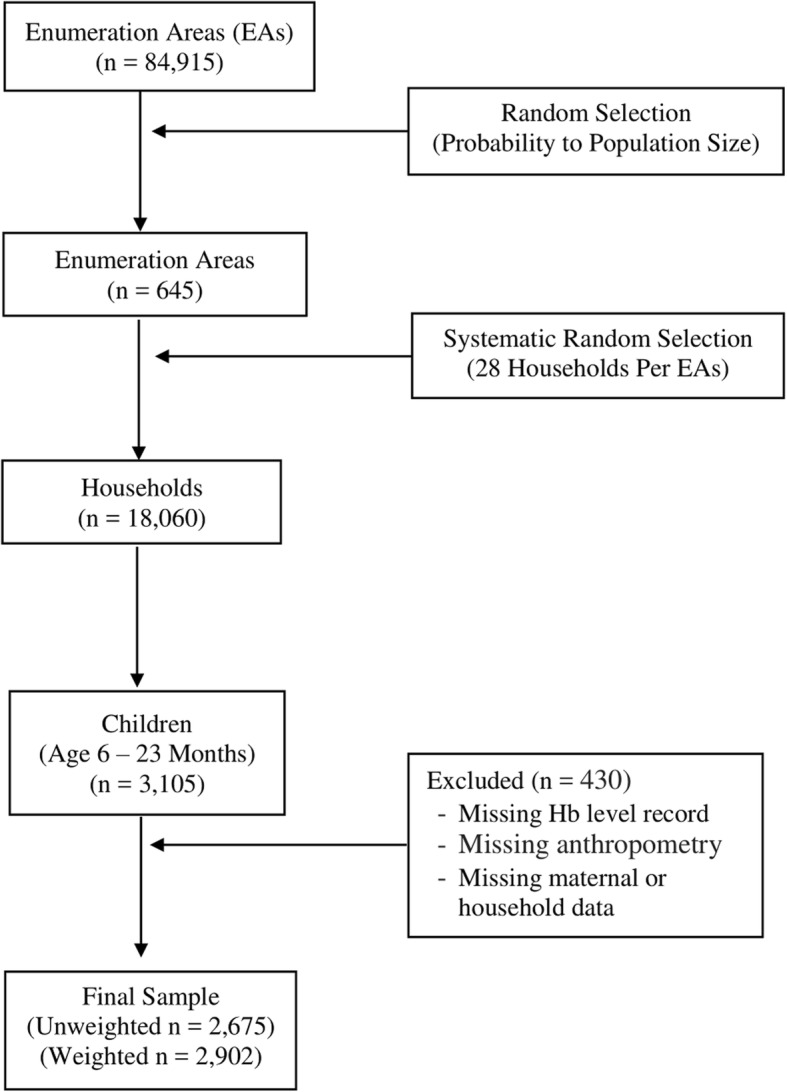
Flow chart of sample selection

EDHS 2016 followed a stratified, two-stage cluster design in sample selection. Census enumeration areas (EAs) were the primary sampling units. The sample included 645 EAs, 202 urban and 443 rural EAs. The secondary sampling units were households. In the second stage of sampling, a fixed number of 28 households were selected from each cluster (EAs), by systematic random sampling. All children in the selected households were included and data were collected on various health and nutrition variables, including Hb level measurement for children aged 6 to 59 months. More information about the methodology of EDHS 2016 can be found in the report of the main findings of the survey [2]. As our interest in this work was on infants and young children, we extracted the data set of only those children aged 6–23 months. We found a total of 3105 children aged 6–23 months. Of these, 430 children with no complete record were excluded from the final dataset. The remaining 2675 children were weighted by their corresponding regional sampling weights, providing a final weighted sample size of 2902 children (Fig. 1).

### Variables and measurements

#### Outcome variable

The main outcome variable was Hb level (g/dL), which is a reliable indicator of anemia at the population level [3]. Blood samples for Hb test were drawn from a finger or a heel prick. The Hb level was determined by battery operated HemoCue®201 analyzers (Sweden) [2]. Then, the Hb measures were adjusted for the altitude of the house of the child. In this analysis, Hb level was used on a continuous scale for better statistical power [15] and as it enables evaluating the relation of the determinants with the full spectrum of Hb level, not just with the state of anemia.

### Explanatory variables

The selection of the explanatory factors was guided by the literature and availability of the variable in the dataset. The variables were categorized into three groups: household, maternal, and child factors.

Household factors: place of residence (urban, rural), region (‘mainly agrarian’: and ‘mainly pastoral’), household wealth category (poor, middle, rich), drinking water sources (improved, unimproved), and toilet facility (improved, unimproved). The household wealth index was calculated by principal component analysis using asset variables collected by the survey and then categorized into tertiles: poor, middle, and rich. Improved sources of drinking water included piped water, bottled water, and protected wells in the compound. Unprotected wells, springs, rivers, ponds, lakes, and dams were grouped as unimproved water sources. Improved household toilet facilities included flush toilets and ventilated pit latrines. Unimproved household toilet facilities were traditional pit latrines.

Maternal factors: body mass index (BMI) (< 18.5, ≥ 18.5 kg/m^2^), anemia status (anemic, not anemic), education status as defined by the highest education level completed (illiterate/none, primary, secondary+), and antenatal care visits (ANC). ANC visits refer to the number of health facility visits the mother attended during the pregnancy of the indexed child and categorized into two groups (< 4, 4+ visits).

Child factors: sex (boy, girl), age (< 12, 12–23 months), birth size (as reported subjectively by the mother of the child, grouped into three categories: large, average, small); and other anthropometry, health, and dietary practice indicators. According to the WHO 2006 criteria [17], Z-score less than − 2 standard deviations (SD) was used to classify children’s nutritional status into stunted (low height-for-age), underweight (low weight-for-age), and wasting (low weight-for-height). History of infection (yes, no) was measured by subjective reporting of the mother or caregiver of the child on whether the child had fever, diarrhea, or cough in the last two weeks preceding the survey. Current breastfeeding status (yes, no), early initiation of breastfeeding within the first one hour after birth (yes, no), deworming in the last six months preceding the survey (yes, no), vitamin A supplement use in the last six months preceding the survey (yes, no), iron supplement use in the last seven days preceding the survey (yes, no), and complementary feeding practices (dietary diversity and meal frequency) were also included. Dietary diversity and meal frequency scores were developed based on the 24 h dietary recall data, which were further categorized into seven food groups: (1) meat, (2) eggs, (3) dairy products, (4) grains, roots, and tubers, (5) legumes and nuts, (6) vitamin-A rich fruits and vegetables, and (7) other fruits and vegetables. According to the WHO criteria, minimum dietary diversity (MDD) is fed from four or more of the above seven food groups and minimum meal frequency (MMF) is met when a child is fed at least three times a day for breastfeeding children and four times a day for non-breastfeeding children [18].

### Statistical analysis

The analysis was done taking into account the complex design of the survey; such that the estimates provided were done based on the weighted data and taking into account the cluster design of the study. Sample weights were applied to compensate for the unequal probability of selection of study participants by region of residence. Small regions were oversampled to ensure data representativeness at regional levels. Thus, following the DHS methodology, sample weights were applied to ensure the data resemble the national population distribution. A detailed explanation of the sampling weighting procedures can be found in the EDHS 2016 report [2]. Bivariable analyses were done to evaluate the relation of each explanatory variable with Hb level. Variables with *P* < 0.25 in the bivariable analyses were included in the final three-stage hierarchical regression analyses, which took into account the relationship among the determinant variables. Thus, three models were constructed, following the approach recommended by Victoria et al. [14]. Statistical significance (*P* ≤ 0.05) of a variable during the hierarchical linear regression analyses was determined at the corresponding model in which the variable of interest was first entered, irrespective its performance in the subsequent model(s). This approach was aimed to avoid the possibility that intermediate variables affect the relation of the distal variables with the outcome variable (Hb level). All data analyses were conducted using STATA version 15, and running “svyset cluster [pw=weight]” command before all analyses.

## Result

In this work, we included a total of 2902 children aged 6–23 months, of which 1359 (46.83%) were boys and 1543 (53.17%) girls. The majority of study participants were from rural areas (89.22%). The mean age (± SD) was 14.01 ± 5.02 months. The majority of children were from middle- and low- income households (67.22%). The mean Hb level (± SD) was 10.00 ± 1.63 g/dL. The overall prevalence of anemia (Hb level < 11 g/dL) among the study population was 71.92%.

Table 1 shows the results of the bivariable analyses of the relation of the household and the maternal factors with Hb level. The household-related factors found significantly associated with a higher mean Hb level were living in urban areas, agrarian regions, and households of high wealth category and improved water supply. Among the maternal characteristics, age, anemia status, education level, and ANC visits were significantly associated with Hb level (*P* < 0.05). Toilet facility and maternal BMI were not significantly associated with Hb level during the bivariate analyses (*P* > 0.05).

**Table 1:** Bivariable analyses of the relation of household and maternal factors with Hb level (g/dL) (*n* = 2902)

Variables		Weighted Frequency (%)	Mean Hb (95%CI)	*P* ^a^
Residence place	Urban	10.78	10.35(10.18, 10.51)	< 0.001
	Rural	89.22	9.95 (9.89, 10.02)	
Region (state)	Pastoral	6.56	9.29(9.05, 9.53)	< 0.001
	Agrarian	93.44	10.05(9.99, 10.11)	
Wealth category	Poor	44.16	9.69 (9.60, 9.79)	< 0.001
	Middle	23.06	10.18 (10.07, 10.28)	
	Rich	32.78	10.28 (10.18, 10.38)	
Toilet facility	Not improved	91.40	9.99 (9.93, 10.05)	0.607
	Improved	8.60	10.05 (9.85, 10.25)	
Water source	Not improved	43.39	9.88 (9.79, 9.97)	0.001
	Improved	56.61	10.08(10.01,10.16)	
Maternal BMI (kg/m^2^)	< 18.5	76.01	10.03 (9.96, 10.10)	0.120
	≥ 18.5	23.99	9.92 (9.80, 10.03)	
Maternal anemia	Not anemic	69.62	10.16 (10.09, 10.23)	< 0.001
	Anemic	30.38	9.63 (9.52, 9.74)	
Education level	Illiterate	61.26	9.91 (9.83, 9.99)	< 0.001
	Primary	31.24	10.03 (9.93, 10.13)	
	Secondary+	7.40	10.52 (10.26, 10.78)	
Antenatal care visits	< 4	65.94	9.93 (9.84, 9.99)	0.003
	4+	34.06	10.11 (10.01, 10.21)	

^a^Independent t-test or one-way ANOVA

The results of the bivariable analyses of the relationship of the dietary and non-dietary child-related factors with Hb level are shown in Table 2. The child factors found significantly associated with Hb level (*P* < 0.05) were sex, age, birth size, height-for-age, weight-for-height, weight-for-age, history of infection, and current breastfeeding status. Early initiation of breastfeeding, deworming medication use in the last six months, iron supplement use in the last seven days, sex, size at birth, MDD, and MMF were not found significantly associated with Hb level (*P* > 0.05). These estimates were, however, crude and less informative, i.e. not adjusted for any covariate factor.

**Table 2:** Bivariable analyses of the relation of child factors with Hb level (g/dL) (*n* = 2902)

Variables		Weighted frequency (%)	Mean Hb (95% CI)	*P* ^a^
Child sex	Boy	46.83	9.91 (9.82, 10.00)	0.006
	Girl	53.17	10.07 (10.00, 10.15)	
Age (months)	< 12	41.63	9.86 (9.77, 9.95)	< 0.001
	12–23	58.37	10.11 (10.03, 10.19)	
Birth size	Small	27.56	9.69 (9.57, 9.81)	< 0.001
	Average	40.84	10.14 (10.05, 10.22)	
	Large	31.60	10.10 (9.99, 10.20)	
Height-for-age	< −2 Z-score	32.22	9.88 (9.77, 9.98)	0.006
	≥ − 2 Z-score	67.78	10.05 (9.98, 10.13)	
Weight-for-age	< −2 Z-score	21.00	9.62 (9.48, 9.76)	< 0.001
	≥ −2 Z-score	79.00	10.10 (10.03, 10.16)	
Weight-for-height	< −2 Z-score	12.88	9.63 (9.48, 9.78)	< 0.001
	≥ −2 Z-score	87.12	10.05 (9.99, 10.12)	
Infection history^b^	No	74.04	10.05 (9.98, 10.12)	0.001
	Yes	25.96	9.81 (9.69, 9.94)	
Current breastfeeding status	No	10.17	9.68 (9.53, 9.83)	< 0.001
	Yes	89.83	9.99 (9.95, 10.03)	
Early breastfeeding initiation	No	10.89	9.92 (9.71, 10.13)	0.558
	Yes	89.11	9.98 (9.91, 10.05)	
Deworming	No	90.57	9.99 (9.93, 10.06)	0.388
	Yes	9.43	10.08 (9.90, 10.26)	
Vitamin A supplement	No	56.35	9.96 (9.88, 10.04)	0.161
	Yes	43.65	10.05 (9.95, 10.14)	
Iron Supplement	No	91.92	10.00 (9.94, 10.07)	0.362
	Yes	8.08	9.90 (9.71, 10.10)	
MDD^c^	No	86.45	9.97 (9.91, 10.04)	0.068
	Yes	13.55	10.14 (10.00, 10.27)	
MMF^d^	No	56.96	9.96 (9.88, 10.04)	0.235
	Yes	43.04	10.03 (9.94, 10.12)	

^a^Independent t-test or one-way ANOVA

^b^Infection defined as (yes, any one of history of cough, diarrhea or fever in the last two weeks preceding the survey)

^c^MDD: Minimum dietary diversity (yes) when a child ate from four or more food groups

^d^MMF: Minimum meal frequency (yes) when a child ate at least three and four times a day for breastfeeding and non-breastfeeding children, respectively

The results of the hierarchical linear regression analyses between the predictor variables and Hb level are shown in Table 3. Residence in agrarian regions was associated with a significantly higher mean Hb level (aβ = 0.64, 95%CI = 0.40–0.88, *P* < 0.001), compared with residence in pastoral regions. Compared with children in the poor wealth category, mean Hb level was significantly higher in those in middle (aβ = 0.42, 95%CI = 0.27–0.58, *P* < 0.001) and rich (aβ = 0.48, 95%CI = 0.33–0.63, *P* < 0.001) categories. Mean Hb level was significantly higher in children of mothers with secondary and above education level (aβ = 0.69, 95%CI = 0.23–1.16, *P* = 0.004), compared with the value of children of illiterate mothers. Compared with children of anemic mothers, Hb level was significantly higher in children of non-anemic mothers (aβ = 0.40, 95%CI = 0.26–0.53, *P* < 0.001).

**Table 3:** Hierarchical linear regression analysis of the relation of household, maternal, and child-related factors with Hb level (g/dL) (*n* = 2902)

Models	Variables		aβ (95% CI)	*P*
Model 1	Residence	Rural	Reference	0.799
		Urban	0.03 (− 0.20, 0.26)	
	Region	Pastoral	Reference	< 0.001*
		Agrarian	0.64 (0.40, 0.88)	
	Wealth category	Poor	Reference	
		Middle	0.42 (0.27, 0.58)	< 0.001*
		Rich	0.48 (0.33, 0.63)	< 0.001*
	Maternal education	Illiterate	Reference	
		Primary	0.00 (−0.14, 0.14)	0.977
		Secondary+	0.69 (0.23, 1.16)	0.004*
Model 2^a^	Water source	Unimproved	Reference	0.846
		Improved	0.01 (−0.12, 0.14)	
	Maternal BMI (kg/m^2^)	< 18.5	Reference	0.744
		≥ 18.5	0.02 (−0.12, 0.17)	
	Maternal anemia	Anemic	Reference	< 0.001*
		Not anemic	0.40 (0.26, 0.53)	
	Antenatal care visits	< 4	Reference	0.567
		≥ 4	0.05 (−0.11, 0.20)	
Model 3^b^	Child sex	Boy	Reference	0.019*
		Girl	0.16 (0.03, 0.30)	
	Child age (months)	< 12	0.72 (0.57, 0.88)	< 0.001
		12–23	Reference	
	Birth size	Small	Reference	
		Average	0.25 (0.08, 0.42)	0.003*
		Large	0.15 (−0.03, 0.33)	0.093
	Height-for-age	< −2 Z-score	Reference	0.630
		≥ −2 Z-score	0.04 (−0.13, 0.21)	
	Weight-for-age	< −2 Z-score	Reference	0.031*
		≥ −2 Z-score	0.22 (0.02, 0.42)	
	Weight-for-height	< −2 Z-score	Reference	0.438
		≥ −2 Z-score	0.09 (−0.13, 0.30)	
	Infection^c^	No	0.18 (0.02, 0.33)	0.025*
		Yes	Reference	
	Currently breastfeeding	No	Reference	0.002*
		Yes	0.28 (0.12, 0.44)	
	Vitamin A supplement	No	Reference	0.021*
		Yes	0.17 (0.06, 0.28)	
	MDD^d^	No	Reference	0.701
		Yes	0.04 (− 0.16, 0.24)	
	MMF^e^	No	Reference	0.034*
		Yes	0.11 (0.05, 0.16)	

^a^Model 2: adjusted for region, wealth category, and maternal education level

^b^Model 3: adjusted for region, wealth category, maternal education level, and maternal anemia

^c^Infection defined as (yes, any one of history of cough, diarrhea or fever in the last two weeks preceding the survey)

^d^MDD: Minimum dietary diversity (yes) when a child ate from four or more food groups

^e^MMF: Minimum meal frequency (yes) when a child ate at least three and four times a day for breastfeeding and non-breastfeeding children, respectively

Being a girl was associated with a significantly higher mean Hb level (aβ = 0.16, 95%CI = 0.03–0.30, *P* = 0.019), compared with being a boy. Mean Hb level was significantly higher in children under 12 months of age than in those above 12 months of age (aβ = 0.72, 95%CI = 0.57–0.88, *P* < 0.001). Children born with an average birth size had a significantly higher mean Hb level (aβ = 0.25, 95%CI = 0.08–0.42, *P* = 0.003), compared with those born with small birth size. Mean Hb level was significantly higher in the non-wasted children (weight-for-age ≥ − 2) (aβ = 0.22, 95%CI = 0.02–0.42, *P* = 0.031) than in the wasted children. Children who had no history of infection in the last two weeks before the survey had a significantly higher mean Hb level (aβ = 0.18, 95%CI = 0.02–0.33, *P* = 0.025). Currently breastfeeding was associated with a significantly higher mean Hb level (aβ = 0.28, 95%CI = 0.12–0.44, *P* = 0.002), compared with not currently breastfeeding. Children who received vitamin A supplementation in the last six months had a significantly higher mean Hb level (aβ = 0.17, 95%CI = 0.06–0.28, *P* = 0.021), compared to those who did not receive the supplementation.

After adjusting for covariate factors, place of residence, household size, water source, maternal BMI, ANC, height-for-age, weight-for-height, MDD, and iron supplementation in the last six months prior to the survey did not demonstrate significant associations with Hb level (*P* > 0.05).

## Discussion

This study was aimed to determine the household, maternal and child factors influencing the Hb level of infants and young children in Ethiopia. We found a high level of anemia. After adjusting for covariates, the household factors found to be associated with Hb level were region of residence and household wealth category. Maternal education level and anemia status were significantly associated with Hb level of children. Sex, birth size, weight-for-age, history of infection, and duration of breastfeeding were the child factors found significantly associated with Hb level.

The high prevalence of anemia we reported was consistent with the report of EDHS 2016, which showed 78, 76, 72, and 66% of anemia prevalence among 6–8, 9–11, 12–17, and 18–23 months old children, respectively [2]. This study showed a lower mean Hb in pastoral regions, compared with the agrarian regions. This might be, in part, due to the high prevalence of malaria in pastoral regions of Ethiopia [19]. Malaria is one of the main risk factors of anemia [5]. Besides, pastoral communities depend on animal milk as a main food source. The low bioavailability of iron in milk could also account for the low Hb level in these communities. Income was a significant and independent predictor of Hb level. Children of poor households had a lower mean Hb level, compared with those of rich households. The result was consistent with previous studies that showed a higher risk of anemia in people with low socioeconomic status [8, 10] and it could be due to the fact that health-enhancing practices and options are often limited among the poor. Children of anemic mothers were more likely to have a lower mean Hb level, compared with those of non-anemic mothers. The result was in agreement with the reports of previous studies [13, 20]. Breast milk is the main source of nutrients during the early stages of life. Thus, the association of maternal anemia with the Hb level of the child could be, in part, due to the low nutrients content of the breast milk of anemic mothers [21].

Weight-for-age was significantly associated with Hb level independent of other factors. The mean Hb of children who were not underweight (weight-for-age ≥ − 2 Z-score) was significantly higher, compared with that of underweight children. Compared with children of low birth weight (small birth size), children of average birth size demonstrated a higher mean Hb level. Previous studies also found child undernutrition associated with anemia [5, 20]. No history of infection, as measured by no cough or fever in the last two weeks prior to the survey, was associated with a significantly higher mean Hb level. The result was in concordance with other studies which reported higher risks of anemia or undernutrition to be associated with infection [5, 22–24].

In this study, iron supplementation and deworming did not demonstrate significant associations with Hb level. At first glance, our findings might appear to conflict with public health recommendations [3, 4]. Iron is essential for the production of red blood cells [3, 21] and Hb level could be presumed to be higher in children who took iron supplements. However, some studies have shown that micronutrient interventions are less effective unless implemented in an integrated approach [25, 26]. The lack of association between these micronutrient interventions and Hb level could be due to some possible reasons like 1) in the DHS program, dose, frequency, and adherence to these interventions were not assessed, albeit they were reported to influence Hb responses to anemia prevention and control interventions [24, 25, 27]. Thus, the lack of accounting for these factors might have influenced our results, 2) the children on the micronutrient supplementations might be the ones who were already anemic and did not yet recover from the state of deficiency, 3) the high level of multiple micronutrient deficiency in Ethiopia [28, 29] could also have influenced the Hb response to iron supplementation as reported by a study in Mexico [25], or 4) it could be due to statistical power inadequacy because the data showed that only a small number of children took the iron supplement as well as the deworming medications.

Our finding was in concordance with previous reports that showed Hb level to be influenced by multiple factors, originating from individual, household, and community levels [5, 10]. Prevention of childhood anemia remains among the major public health agendas in most of the developing countries [1], including Ethiopia [11]. The WHO has outlined essential public health and nutrition interventions to reduce the burden of childhood anemia [3, 21]. Most of the WHO anemia prevention and control recommendations have been incorporated in the Ethiopians National Nutrition Programs and Strategies [11], albeit the implementation has been sub-optimal, fragmented, and mainly focused on addressing the immediate causes [2, 11]. Thus, it stands important for policy makers in Ethiopia to enhance the implementation of the existing anemia prevention and control interventions in a multi-sectoral and comprehensive approach. The determinants of Hb level, particularly the contextual factors, vary significantly across geographical regions. The relative contribution of the determinants to the burden of anemia is also contextual [6]. For example, recent reports have shown that the contribution of iron deficiency to the burden of anemia in developing countries is not as high as the widely held presumption that it accounts for almost 50% of the anemia burden [6]. Thus, it is worthy of conducting further studies considering the geospatial variations in determinants of Hb level and also design locally sensitive interventions.

One of the strengths of this study was the use of a nationally representative sample and objectively measured biomarkers: Hb level, weight, and height. The use of multilevel analysis scheme which took into account the hierarchical nature of the relationships among the various influences of Hb level might have avoided the nullifying effect of the intermediate factors on the association of the distal factors and Hb level. The inclusion of various explanatory factors (child, maternal, and household variables) might have improved the comprehensiveness of the study and enabled adjustment for various potential confounding variables. However, this study has important limitations. First, the design of the study, cross-sectional, precludes establishing temporal relations and making causal inferences. Second, the collection of data on some of the variables based on respondents’ memory of past events might have introduced recall bias. Third, the lack of data on intestinal helminths, reported to influence Hb level, could be another limitation of this study. Fourth, we did not consider hereditary anemias in the study due to the lack of data in the dataset we used. However, it is less likely that our findings were affected by the lack of inclusion of hereditary anemias because the incidence of hereditary anemias is generally low in Ethiopia [30]. Fifth, we did not account for the effects of dose, duration, and adherence of deworming, iron, and vitamin A supplementation, which might have biased our findings. Despite these limitations, we believe the findings of this study could serve as evidence basis for further studies on the determinants of Hb level of children in Ethiopia.

## Conclusion

Hb level of infants and young children in Ethiopia was found to be influenced by various household, maternal and child related factors. Designing and scaling up comprehensive nutrition interventions, with due emphasis on the multifactorial nature of Hb, may represent a potential consideration to reduce the burden of anemia in Ethiopia.

## Abbreviations

ANC: Antenatal care
aβ: Adjusted β
BMI: Body mass index
CI: Confidence interval
DHS: Demographic and health surveys
EDHS: Ethiopian demographic and health survey
Hb: Hemoglobin
MDD: Minimum dietary diversity
MMF: Minimum meal frequency
SD: Standard deviation
USAID: United States Agency for International Development
WHO: World Health Organization
